# Distances between bony landmarks and adjacent nerves: anatomical factors that may influence retractor placement in total hip replacement surgery

**DOI:** 10.1186/s13018-016-0365-2

**Published:** 2016-03-16

**Authors:** Ta-I Wang, Hui-Yi Chen, Chun-Hao Tsai, Horng-Chaung Hsu, Tsung-Li Lin

**Affiliations:** Department of Orthopedics, China Medical University Hospital, China Medical University, Taichung, 40447 Taiwan; Department of Radiology, China Medical University Hospital, China Medical University, Taichung, 40447 Taiwan; Graduate Institute of Clinical Medicine, School of Medicine, China Medical University, Taichung, 40447 Taiwan

**Keywords:** Distances, Bony landmarks, Adjacent nerves, Magnetic resonance imaging, Total hip replacement, Body height

## Abstract

**Background:**

Retractor placement is a leading cause of intraoperative nerve injury during total hip replacement (THR) surgery. The sciatic nerve, femoral nerve, and superior gluteal nerve are most commonly affected. This study aimed to identify the distances from bony landmarks in the hip to the adjacent nerves on magnetic resonance imaging (MRI) and the associations between anatomical factors and these distances that would guide the placement of retractors during THR surgery, in order to minimize the risk of nerve injury.

**Methods:**

We reviewed hip MRIs of 263 adults and recorded the distances from (1) the anterior acetabular rim to the femoral nerve; (2) the superior acetabular rim to the superior gluteal nerve; (3) the posterior acetabular rim to the sciatic nerve; and (4) the greater trochanter to the sciatic nerve. The effects of anatomical factors (i.e., gender, age, body height, body mass index (BMI), pelvic width, and acetabular version and morphology) on these distances were analyzed.

**Results:**

Distances from bony landmarks to adjacent nerves (in cm) were 2.06 ± 0.44, 2.23 ± 0.28, 1.94 ± 0.81, and 4.83 ± 0.26 for the anterior acetabular rim, superior acetabular rim, posterior acetabular rim, and greater trochanter, respectively, and were shorter in women than in men (*P* < 0.001). Multivariate analysis identified body height as the most influential factor (*P* < 0.001). Linear regression demonstrated a strong positive linear correlation between body height and these distances (Pearson’s *r* = 0.808, 0.823, 0.818, and 0.792, respectively (*P* < 0.001)).

**Conclusions:**

The distances from bony landmarks to adjacent nerves provide useful information for placing retractors without causing nerve injury during THR surgery. Shorter patients will have shorter distances from bony landmarks to adjacent nerves, prompting more careful placement of retractors.

## Background

The incidence of nerve injury in primary total hip replacement (THR) surgery ranges from 0.17 to 3.7 % [1–4]. Around 80 % of patients who sustain a THR-related nerve injury have persistent neurologic dysfunction, including paraesthesia, neuropathic pain, or motor weakness [5]. Such injuries have severe effects on patients’ prognoses and reduce their quality of life [6].

The most commonly identifiable causes of intraoperative nerve injuries were leg lengthening, heat generated during polymerization, direct nerve encasement, trauma caused by instrumentation, or inappropriate positioning of retractors [4, 7–15]. In order to maintain a clear surgical field during THR surgery, retractors are placed around the acetabulum and femur. During THR, the nerves (most commonly the sciatic, femoral, and superior gluteal nerves) may be contused, compressed, or penetrated by the tips of retractors [2, 3, 16–18]. In all approaches to the hip, the sciatic nerve lies directly under the greater trochanter and could be injured by deep insertion of the trochanteric retractor [19, 20].

Prevention is the first principle in managing nerve injuries associated with THR surgery and requires complete awareness of the anatomy of the acetabulum and proximal femur and adequate retractor placement. Female gender is the most well-established risk factor for iatrogenic nerve injury during THR [13, 21–23]. Some hypothesize that the increased risk in women is due to smaller stature or reduced muscle mass as compared with men [22, 23]. Other investigators have shown a relationship between safe distance from the superior gluteal nerve and anatomical factors, such as body height, with varying results [24–26]. The female pelvis is on average were significantly but proportionally smaller than the male pelvis in all measurements except pelvic width [27], but the relationship between nerve position and pelvic width was still unclear; however, the effects of these anatomical factors on distances from bony landmarks to adjacent nerves, such as age, body mass index (BMI), and pelvic width have not been elucidated.

This study aimed to determine [1] the distances between important bony landmarks in the hip and the adjacent nerves in adults on magnetic resonance imaging (MRI) and [2] the significance of associations between anatomical factors (i.e., gender, age, body height, BMI, pelvic width, and acetabular version and morphology) and these distances that would guide the placement of retractors in order to minimize the risk of nerve injuries during THR surgery.

## Methods

### Patients

After obtaining institutional review board approval (China Medical University Hospital 104-Research Ethics Committee 3-043), we conducted a retrospective review of 263 consecutive hip MRI studies (avascular necrosis (*N* = 147), osteoarthritis (*N* = 52), femoroacetabular impingement (*N* = 38), labral tear (*N* = 26)) performed on 263 patients between 20 and 80 years of age during a 9-year interval (January 2002 to December 2010). All studies had a written report submitted by a musculoskeletal radiologist (HYC) at our institution. All cases were unilateral pathological hips. The age of each patient at the time of the study was noted. From the medical records, we collected data on anatomical factors, including gender, body height, and body weight. BMI was calculated as weight in kilograms divided by height in meters squared (kg/m^2^). The pelvic width, defined as the distance between the anterior superior iliac spines [28, 29], was reviewed on all supine anteroposterior pelvis radiographs. Acetabular morphologies, such as protrusio acetabuli [30] and standard superolateral osteoarthritis, were recorded. The study group included 140 men and 123 women with an average age of 48.71 years (range 20–76), a mean height of 165.87 cm (range 140–186), and a mean BMI of 26.67 (range 16.8–44.8).

### Magnetic resonance imaging

Patients were scanned with a 1.5 Tesla Signa MRI scanner (General Electric Medical Systems, Milwaukee, WI) in the supine position, with both lower extremities straight and knees extended. Imaging studies focused on the entire pelvis from the iliac crest to below the lesser trochanter. T1-weighted images in the axial, sagittal, and coronal planes with slice thickness of 2 mm were selected for analysis. The distances from the three acetabular bony landmarks and one trochanteric bony landmark to adjacent nerves were measured on each bilateral hip MRI.

**Fig. 1:**
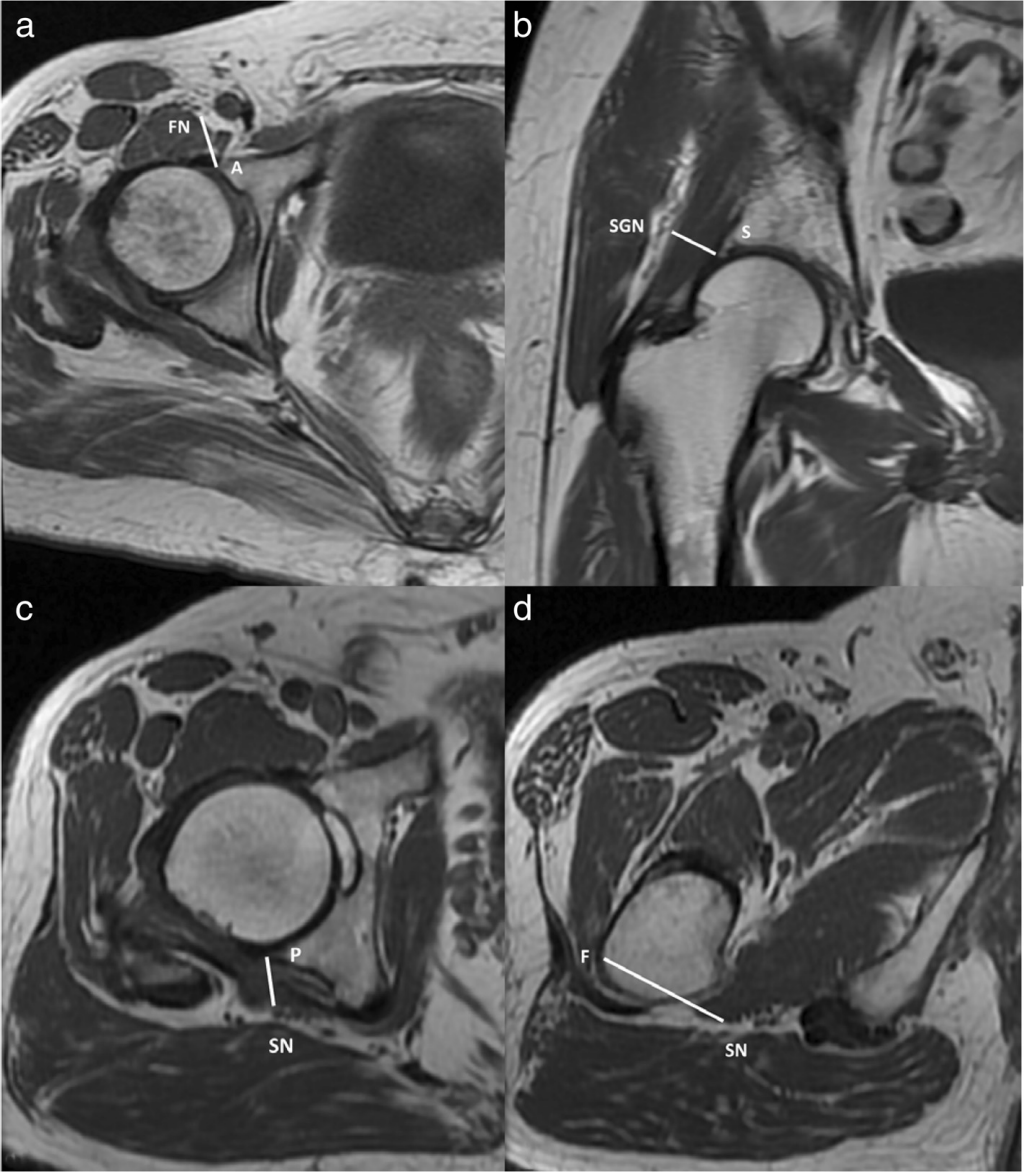
Magnetic resonance images demonstrating the views used for measuring distances from bony landmarks to adjacent nerves: **a** anterior acetabular rim (*A*) to the femoral nerve (*FN*); **b** superior acetabular rim (*S*) to the superior gluteal nerve (*SGN*); **c** posterior acetabular rim (*P*) to the sciatic nerve (*SN*); **d** greater trochanter (*G*) to the sciatic nerve (*SN*)

**Fig. 2:**
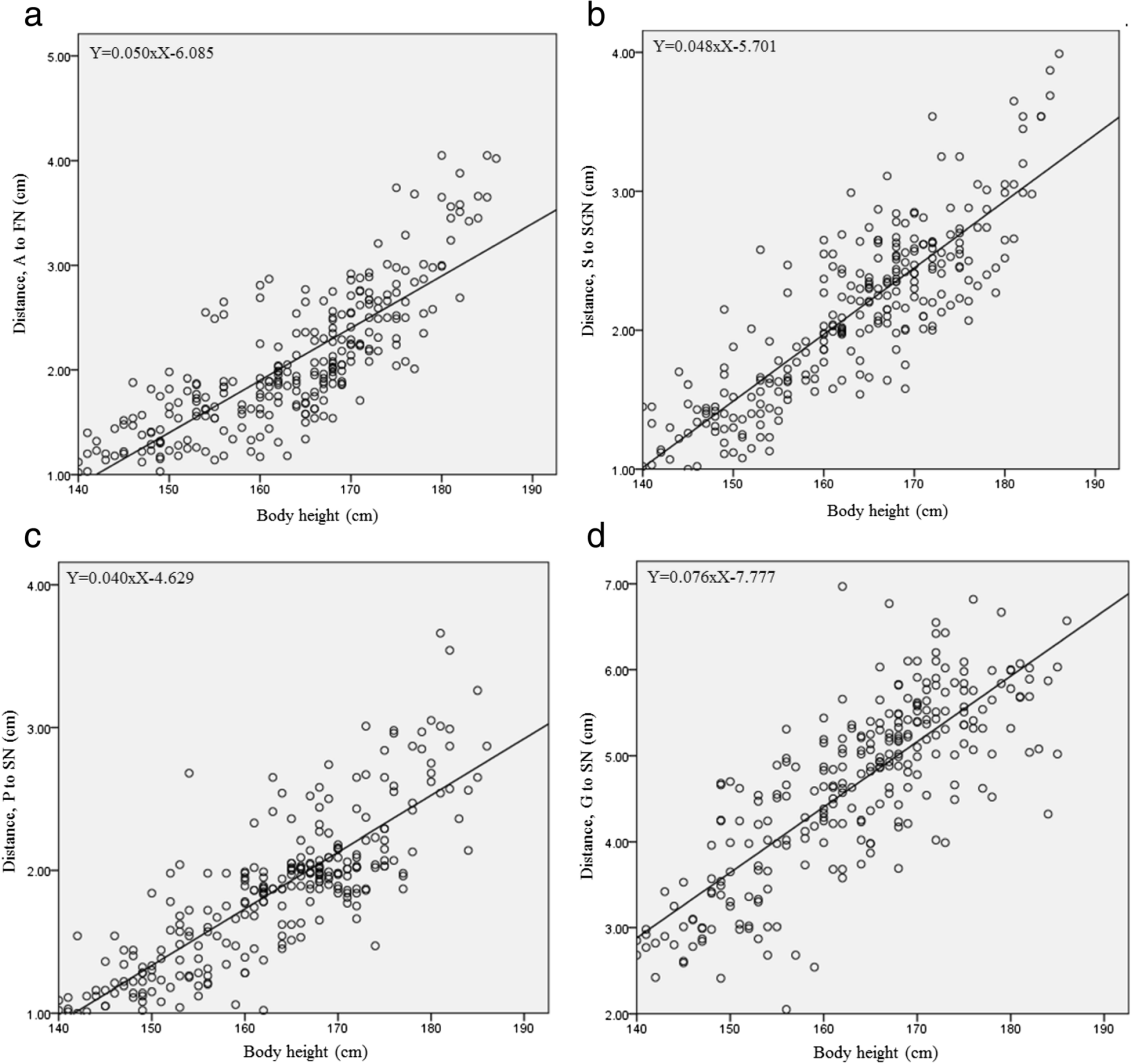
The simple linear regression model describing the relationship between body height and distances from **a** anterior acetabular rim (*A*) to the femoral nerve (*FN*); **b** superior acetabular rim (*S*) to the superior gluteal nerve (*SGN*); **c** posterior acetabular rim (*P*) to the sciatic nerve (*SN*); **d** greater trochanter (*G*) to the sciatic nerve (*SN*)

The distances (in centimeters (cm)) were measured using a digital caliper tool within INFINITT’s Picture Archiving and Communications System (PACS) as follows (Table 1): (1) the anterior acetabular rim at the 3 o’clock position on the right side, or the 9 o’clock position on the left side, to the femoral nerve (Fig. 1a), (2) the superior acetabular rim at the 12 o’clock position, to the superior gluteal nerve (Fig. 1b), (3) the posterior acetabular rim at the 9 o’clock position on the right side, or the 3 o’clock position on the left side, to the sciatic nerve (Fig. 1c), and (4) the most lateral point of the greater trochanteric ridge to the sciatic nerve (Fig. 1d). The acetabular version was measured on each MRI [31]. One musculoskeletal radiologist (HYC) and two orthopedic surgeons (TIW, CHH) recorded all measurements independently, and the mean was used for data analysis. Intra- and interobserver reliability was tested for each measurement using the intraclass correlation coefficient (ICC) for continuous value measurements (Table 1).

**Table 1:** Descriptions and reliability/reproducibility of the four distances between bony landmarks and adjacent nerves

Distances	Description	Intrarater ICC	Interrater ICC
A acetabular rim to FN (Fig. 1a)	In axial images, the cut of largest diameter of the femoral head was chosen after tracing nearby cuts. The anterior acetabular rim at the 3 o’clock position on the right side, or the 9 o’clock position on the left side, was defined as “anterior acetabular rim.” The closest distance between this point and the femoral nerve was measured	0.92 (0.82–0.97)	0.90 (0.82–0.96)
S acetabular rim to SGN (Fig. 1b)	In coronal images, the cut of largest diameter of the femoral head was chosen after tracing nearby cuts. The superior acetabular rim at the 12 o’clock position was defined as “superior acetabular rim.” The closest distance between this point and the superior gluteal nerve was measured	0.97 (0.88–0.98)	0.91 (0.86–0.93)
P acetabular rim to SN (Fig. 1c)	In axial images, the cut of largest diameter of the femoral head was chosen after tracing nearby cuts. The posterior acetabular rim at the 9 o’clock position on the right side, or the 3 o’clock position on the left side, was defined as “posterior acetabular rim”. The closest distance between the point and the sciatic nerve was measured	0.92 (0.85–0.94)	0.93 (0.88–0.96)
G to SN (Fig. 1d)	In axial images, the most lateral point of the greater trochanteric ridge was chosen after tracing nearby cuts. The closest distance between this point and the sciatic nerve was measured	0.97 (0.92–0.99)	0.96 (0.90–0.98)

Values are expressed as mean, with 95 % CI in parentheses

*A* anterior, *FN* femora nerve, *S* superior, *SGN* superior gluteal nerve, *P* posterior, *SN* sciatic nerve, *G* greater trochanter, *ICC* intraclass correlation coefficient

### Statistical analysis

Statistical analyses were performed using SPSS for Windows, version 21.0 (SPSS Inc., Chicago, IL, USA). Continuous data are presented in the form of mean ± standard deviation. Groups were compared using a *t* test for independent samples. The effects of gender, age, body height, BMI, pelvic width, and acetabular version and morphology on each measurement were evaluated by multivariate linear regression analysis. The coefficient of determination, *R*^2^, was used to check the goodness of fit of the statistical models and as a measure of how much of the original uncertainty in the data was explained by the multivariate analysis. *R*^2^ varied between 0 and 1, with 0 indicating no benefit gained by applying multivariate analysis and 1 indicating benefit. The correlation between the most influential anatomical factor and distance measurements was analyzed using Pearson’s correlation coefficient. Meanwhile, Games-Howell post hoc analysis was used to test for significant differences in the most influential anatomical factor between the mean distances. Statistical significance was set at *P* < 0.05.

## Results

The prevalence of acetabular retroversion in this study was 7.8 % (41/526 hips; avascular necrosis (*N* = 12), osteoarthritis (*N* = 10), femoroacetabular impingement (*N* = 6), labral tear (*N* = 2), normal hip (*N* = 11)). There was no significant difference between the retroverted and anteverted acetabulum in distances (in cm) between the aforementioned landmarks and the adjacent nerves (2.08 ± 0.51 vs. 2.05 ± 0.76, *P* = 0.745; 2.05 ± 0.33 vs. 2.26 ± 0.87, *P* = 0.334; 1.86 ± 0.23 vs. 1.98 ± 0.18, *P* = 0.637; 4.75 ± 0.38 vs. 4.86 ± 0.66, *P* = 0.126, respectively).

Protrusio acetabuli accounted for 6.8 % (36/526 hips), while standard superolateral osteoarthritis accounted for 7.8 % (41/526 hips). The difference in the distances between the bony landmarks and the adjacent nerves among the two groups was not statistically significant (1.99. ± 0.14 vs. 2.03 ± 0.22, *P* = 0.421; 2.41 ± 0.81 vs. 2.02 ± 0.56, *P* = 0.337; 1.71 ± 0.37 vs. 1.88 ± 0.64, *P* = 0.411; 4.01 ± 0.58 vs. 4.13 ± 0.75, *P* = 0.287, respectively).

The distances between the bony landmarks and the adjacent nerves of the pathologic hip were 2.06 ± 0.44, 2.23 ± 0.28, 1.94 ± 0.81, and 4.83 ± 0.26, respectively, while of the normal hip were 2.05 ± 0.23, 2.25 ± 0.45, 1.96 ± 0.24, and 4.79 ± 0.88. There was no significant difference between the pathologic and normal hips in distances (*P* = 0.831). We chose the distances to the pathologic hips for analysis because they were reflective of the anatomical reality in patients actually undergoing THR.

There was a significant gender difference with regard to distances from bony landmarks to adjacent nerves in relation to body height (*P* < 0.001; Table 2), but not to age, BMI, or pelvic width (*P* = 0.471, 0.511, and 0.227, respectively). Because a gender difference in body height might account for differences in distances from bony landmarks to adjacent nerves, multivariate analysis was performed.

**Table 2:** Gender differences in age, BH, BMI, PW and distances between bony landmarks and adjacent nerves

	Men (*n* = 140)	Women (*n* = 123)	*P* value
Age (years)	49.23 ± 10.11	51.54 ± 12.60	0.471
BH (cm)	170.83 ± 8.65	158.06 ± 7.42	<0.001
BMI (kg/m^2^)	26.52 ± 4.19	24.92 ± 4.26	0.511
PW (cm)	26.57 ± 1.65	26.81 ± 1.67	0.227
Distances (cm)			
A acetabular rim to FN	2.35 ± 0.37	1.74 ± 0.82	<0.001
S acetabular rim to SGN	2.50 ± 0.54	1.92 ± 0.71	<0.001
P acetabular rim to SN	2.29 ± 0.28	1.59 ± 0.61	<0.001
G to SN	5.32 ± 0.25	4.22 ± 0.22	<0.001

Data are presented as mean ± standard deviation

*BH* body height, *BMI* body mass index, *PW* pelvic width, *A* anterior, *FN* femora nerve, *S* superior, *SGN* superior gluteal nerve, *P* posterior, *SN* sciatic nerve, *G* greater trochanter

The results are shown in Table 3. The total effect of these anatomical factors, *R*^2^, on the anterior acetabular rim, superior acetabular rim, posterior acetabular rim, and greater trochanter was 0.578, 0.660, 0.711, and 0.610, respectively. The distances from bony landmarks to adjacent nerves correlated with body height (*P* < 0.001) but not with gender, age, BMI, or pelvic width. The linear regression equations predicting bony landmarks-to-adjacent nerves distances from body height were as the below formulas:

- anterior acetabular rimto the femoral nerve (cm) = 0.050 × body height (cm)–6.085 (Fig. 2a)
- superior acetabular rim to the superior gluteal nerve (cm) = 0.048 × body height (cm)–5.701 (Fig. 2b)
- posterior acetabular rim to the sciatic nerve (cm) = 0.040 × body height (cm)–4.629 (Fig. 2c)
- greater trochanter to the sciatic nerve (cm) = 0.076 × body height (cm)–7.777 (Fig. 2d)

**Table 3:** Multivariate analyses on distances from bony landmarks to adjacent nerves

Distances	B estimate	SE	*P* value	*R* ^2^
A acetabular rim to FN				0.578
Intercept	−4.938	0.588		
Gender	−0.023	0.049	0.689	
Age (years)	0.002	0.001	0.233	
BH (cm)	0.052	0.006	<0.001	
BMI (kg/m^2^)	−0.004	0.003	0.662	
PW (cm)	−0.005	0.002	0.992	
S acetabular rim to SGN				0.660
Intercept	−4.418	0.527		
Gender	−0.059	0.039	0.197	
Age (years)	0.001	0.003	0.794	
BH (cm)	0.042	0.002	<0.001	
BMI (kg/m^2^)	−0.002	0.005	0.619	
PW (cm)	−0.001	0.001	0.431	
P acetabular rim to SN				0.711
Intercept	−4.310	0.452		
Gender	−0.065	0.039	0.096	
Age (years)	0.000	0.001	0.843	
BH (cm)	0.040	0.002	<0.001	
BMI (kg/m^2^)	−0.002	0.003	0.649	
PW (cm)	−0.001	0.001	0.393	
G to SN				0.610
Intercept	−6.819	1.014		
Gender	−0.133	0.084	0.129	
Age (years)	0.000	0.001	0.948	
BH (cm)	0.076	0.005	<0.001	
BMI (kg/m^2^)	0.002	0.004	0.693	
PW (cm)	−0.002	0.003	0.402	

*BH* body height, *BMI* body mass index, *SE* standard error, *R* ^*2*^ coefficient of determination, *A* anterior, *FN* femoral nerve, *S* superior, *SGN* superior gluteal nerve, *P* posterior, *SN* sciatic nerve, *G* greater trochanter

These formulas predicted that shorter patients (especially a patient shorter than 150 cm) have shorter distances from bony landmarks to adjacent nerves (Table 4). These distances were linearly positively correlated with body height (Pearson’s *r* = 0.808, 0.823, 0.818, and 0.792, in anterior acetabular rim, superior acetabular rim, posterior acetabular rim, and greater trochanter, respectively; *P* < 0.001).

**Table 4:** Distances between bony landmarks and adjacent nerves based on body height

	Body height (cm)					
Distance (cm)	<150 (*n* = 35)	150–159 (*n* = 49)	160–169 (*n* = 99)	170–179 (*n* = 62)	>180 (*n* = 18)	*P* value
A acetabular rim to FN	1.32 ± 0.21	1.66 ± 0.35	1.97 ± 0.36	2.58 ± 0.38	3.49 ± 0.39	<0.001
S acetabular rim to SGN	1.36 ± 0.24	1.59 ± 0.32	2.24 ± 0.33	2.53 ± 0.31	3.21 ± 0.47	<0.001
P acetabular rim to SN	1.19 ± 0.15	1.49 ± 0.31	1.93 ± 0.29	2.16 ± 0.35	2.83 ± 0.38	<0.001
G to SN	3.21 ± 0.58	3.85 ± 0.75	4.88 ± 0.62	5.50 ± 0.58	5.66 ± 0.52	<0.001

Data are presented as mean ± standard deviation

*A* anterior, *FN* femora nerve, *S* superior, *SGN* superior gluteal nerve, *P* posterior, *SN* sciatic nerve, *G* greater trochanter

## Discussion

Retractor placement is a leading cause of intraoperative nerve injury during THR surgery [2, 4]. The sciatic nerve, femoral nerve, and superior gluteal nerve are most commonly affected. In this study, reference values for safe distances were established using in vivo imaging, MRI, in adult hips. We also assessed the effect of anatomical factors including gender, age, body height, BMI, pelvic width, and acetabular version and morphology on these distances.

Shubert et al. have described the appropriate positions of acetabular retractors and the distances between retractors and the neurovascular bundles by using both computed tomography (CT) images and cadaveric specimens [9]. He studied the distances from five common acetabular retractor positions to the adjacent neurovascular structure and identified the anterior inferior iliac spine (AIIS) as the farthest point from the femoral neurovascular bundle and the safest anterior acetabular retractor position. Although we retrospectively measured distances from bony landmarks to adjacent nerves on hip MRI instead of actual distances from retractors to adjacent nerves intra-operatively, the three acetabular bony distances from landmarks to adjacent nerves measured in our study were similar to those reported by Shubert et al. [9]. Moreover, the sample size in our study was larger (263 MRIs vs. 32 CTs and 16 cadavers). In addition, the MRIs in our study were used to assess structurally pathologic hips, reflective of the anatomical reality in patients actually undergoing THR. All distances were independently measured by three physicians. Therefore, the results of this study should provide more reliable reference data.

The femoral nerve, which arises from the L2–L4 nerve roots, passes through the psoas muscle and travels between the psoas and the iliacus to enter the thigh as the lateral most structure of the femoral triangle. This study found that the distance from the anterior acetabular rim to the femoral nerve was 2.06 ± 0.44 cm. This distance is similar to the mean distance of 2.2 cm between the femoral nerve and the anterior capsule reported by Davis et al. [32] and measured on MRI of 11 healthy hips. Slater et al. [33] stated that placement of the anterior acetabular retractor was the surgical maneuver causing the greatest pressure adjacent to the femoral nerve during THR surgery; however, the femoral nerve is well protected from maneuvers involving the acetabulum by being located behind the iliopsoas muscle on the opposite side of the hip joint. This may explain the relative rarity of femoral nerve lesions and suggests that most femoral nerve lesions are caused by the incorrect use of retractors or instruments during hip surgery [12, 34]. Therefore, a clear understanding of the anatomy of the femoral triangle as well as accurate placement of the anterior acetabular retractor can minimize the incidence of this complication [35].

The superior gluteal nerve, which arises from the L4–S1 nerve roots, exits through the sciatic notch to supply the gluteus medius, gluteus minimus, and tensor fascia lata. It travels deep to the gluteus medius, but remains superficial to the gluteus minimus. The superior gluteal nerve is most commonly injured during the anterolateral (Watson-Jones), lateral (Hardinge), or transtrochanteric approach when the 3–5-cm “safe area” proximal to the tip of greater trochanter is violated [14, 24, 36–38]. This study found that the distance from the superior acetabular rim to the superior gluteal nerve was 2.23 ± 0.28 cm—shorter than the distance described in the literature; however, the distance we measured was from the superior bony rim of the acetabulum rather than from the tip of the greater trochanter as measured in previous studies [14, 24, 38].

The sciatic nerve, which arises from the L4–S3 nerve roots, is composed of independent tibial and fibular divisions. These two nerve trunks enveloped by a common fascial sheath can be distinguished at their origins, and they leave the pelvis through the greater sciatic foramen below the piriformis. The sciatic nerve was involved in over 90 % of the 53 nerve injuries (3000 cases) reported by Schmalzried et al. [5]. This study found that the distance from the posterior acetabular rim to the sciatic nerve was 1.94 ± 0.81 cm and was shorter than the distances from other bony landmarks to adjacent nerves. This may explain why sciatic nerve injury is so common during THR surgery [2, 4, 15, 39]. Because of its proximity to the sciatic nerve, the posterior acetabular retractor should be placed carefully and adjusted as needed during acetabular preparation.

The placement of the trochanteric retractor during femoral preparation in the anterolateral approach places the sciatic nerve at greater risk. In the lateral decubitus position and with the femur externally rotated, the sciatic nerve is found directly posterior to the greater trochanter. When inserting an elevating trochanteric retractor, its tip slips between the greater trochanter and the sciatic nerve, which, due to this special topographic relationship, can be injured by deep insertion of the retractor [19, 20]. The fact that the fibular division is more lateral may increase its vulnerability to trauma [4, 40]. Schmalzried et al. found that 94 % of the sciatic nerve injuries in their study involved the fibular division, while the tibial division was only rarely involved by itself (2 % of cases) [5]. In magnetic resonance neurography (MRN) studies of nine patients with sciatic nerve palsy related to THR surgery, Wolf et al. reported more frequent involvement of the fibular division than of the tibial division of the sciatic nerve [8]. Moreover, the fibular division is more tethered at the sciatic notch and the fibular neck, thus making it less tolerant to tension during acute stretching and more susceptible to damage than is the tibial division [8, 10, 41–43]. In addition, the fibular division is composed of fewer and larger funiculi that are more tightly packed together with less connective tissue than is found in the tibial division. This predisposes the fibular division to mechanical injury because the neural elements have less room for displacement and dissipation of the force to intervening connective tissue. Consequently, the potential for recovery of the fibular division is more restricted than is that of the tibial division [10]; however, there is a paucity of literature on how close the tip of the trochanteric retractor can be to the most lateral part of sciatic nerve during preparation of the femur. This study found that the distance from the greater trochanter to the sciatic nerve was 4.79 ± 0.88 cm and was larger than the distance of other bony landmarks to adjacent nerves; however, it is a useful reference value when placing the retractor deeply into the greater trochanter, especially during minimally invasive approaches, which make femoral exposure difficult and may cause problems [44–46].

Female gender is the best-established risk factor for nerve injury during THR surgery, with multiple studies reporting that at least 74 % of these events occur in women [4, 13, 21–23]. This study found that all distances from bony landmarks to adjacent nerves were significantly shorter in women and that this was a likely explanation of why most intraoperative neurologic injuries occur in women [13, 21–23]. Some hypothesize that the increased risk in women is due to variation in neurovascular anatomy, shorter stature, or reduced muscle mass compared with men, while others believe that the increased risk is related to the prevalence of developmental dysplasia of the hip (DDH) [2, 10, 22, 23, 47, 48]; however, based on the multivariate analyses of this study, body height was a major contributing factor to these reduced distances. The other factors including gender, age, BMI, and pelvic width showed no significant relation to these distances. Thus, shorter stature of the women studied may account for female gender being a risk factor for neurologic injury during THR surgery. Unfortunately, only a few studies have focused on the relationship between body height and safe distances from the superior gluteal nerve, and these studies have had contradictory results [24–26]. To our best knowledge, our study was the first to evaluate the effects of body height, gender, age, BMI, and pelvic width on distances from bony landmarks to adjacent nerves of hips as measured on MRI. Moreover, linear regression analysis demonstrated a positive correlation between the distances and body height. Using the formulas mentioned in the results section, hip surgeons can now use body height to estimate safe distances. The results are very encouraging: shorter patients have shorter distances from bony landmarks to adjacent nerves, and surgeons need to take this into account when placing retractors during THR surgery.

In this study, the sciatic nerve seems to be more vulnerable in the retroverted acetabulum (1.86 ± 0.23 vs. 1.98 ± 0.18, *P* = 0.637), while the femoral nerve seems to be more vulnerable in the anteverted acetabulum (2.08 ± 0.51 vs. 2.05 ± 0.76, *P* = 0.745), but there was no statistical significance. Moreover, there was no significant difference between the protrusion deformity and standard superolateral osteoarthritis in distances (*P* = 0.448). We conclude that the acetabular version and morphology do not affect the distance between the bony landmarks and the adjacent nerves.

A number of limitations to our study are worth highlighting. First, our distances were measured on MRIs, not intraoperatively. Each MRI was taken with the patient in a supine position. Most surgeons performed THR surgery in the lateral decubitus position, and anatomic relationships may differ with positioning. Second, we did not take the dimensions of retractors into consideration, and the distances between tips of retractors and adjacent nerves intraoperatively would be shorter. Third, we chose pelvic width as an anatomical factor instead of pelvic type. We found in a review of the literature that there was no evidence supporting the relationship between nerve position and pelvic type [49]. Fourth, instead of measuring distances on 2D MRI scans, it would be more reliable to make 3D reconstructions in software Mimics® (Materialise, Leuven, Belgium) of the soft tissue of interest, i.e., the three nerves, and to relate this course/distance to the 3D bony landmarks with the use of an X, Y, Z axis system. This provides a platform for further evaluation. Furthermore, all subjects were unrelated ethnic Han Chinese recruited from the authors’ institution. It would be interesting to conduct independent studies in other ethnic populations for comparison.

By measurement on hip MRIs of a large sample of adults, this study revealed safe distances from bony landmarks to adjacent nerves that could be used for placement of retractors to avoid nerve injury during THR surgery. During acetabular preparation, surgeons strive to apply the retractor tips as close to the bony rim as possible (leaving 1.94 to 2.23 cm between bony landmarks and the adjacent nerves) to avoid nerve injury. The positions of the acetabular retractors should be checked periodically to ensure that migration has not occurred, given the close proximity of adjacent nerves. Care should be taken in placing the trochanteric retractor posterior to the greater trochanter during femoral preparation. If too deep, placement may endanger the sciatic nerve, especially during minimally invasive approaches to the hip. The anatomical factor of body height must be taken into consideration for retractor placement during THR surgery. These considerations are particularly important in shorter patients, in whom distances from bony landmarks to adjacent nerves will be shorter.

## Conclusions

The distances from bony landmarks to adjacent nerves provide useful information for placing retractors without causing nerve injury during THR surgery. Shorter patients will have shorter distances from bony landmarks to adjacent nerves, prompting more careful placement of retractors.

## Abbreviations

AIIS: anterior inferior iliac spine
BMI: body mass index
CT: computed tomography
DDH: developmental dysplasia of the hip
ICC: intraclass correlation coefficient
MRI: magnetic resonance imaging
MRN: magnetic resonance neurography
*R*^2^: coefficient of determination
THR: total hip replacement
